# Calycosin targets the MAPK-EGR1 signaling axis to inhibit enterovirus 71 replication in cells and human colonic organoids

**DOI:** 10.1080/21505594.2026.2711500

**Published:** 2026-08-05

**Authors:** Yue Liu, Jiale Wang, Xin Chen, Mengli Wu, Xiangqian Zhang, Yan-Heng Zhou

**Affiliations:** a Shaanxi Engineering and Technological Research Center for Conversation and Utilization of Regional Biological Resources, College of Life Sciences, Yan’an University, Yan’an, Shaanxi, China; b Department of Pathogenic Biology, School of Basic Medical Sciences, Gannan Medical University, Ganzhou, Jiangxi, China; c School of Medical Technology and Information Engineering, Zhejiang Chinese Medical University, Hangzhou, Zhejiang, China; d Engineering Research Center of Microbial Resources Development and Green Recycling, University of Shaanxi Province, Yan’an University, Yan’an, Shaanxi, China

**Keywords:** EV71, MAPK, EGR1, viral replication, cell death, Calycosin, organoids

## Abstract

The downstream effectors that mediate the pro‑viral function of the MAPK pathway during enterovirus 71 (EV71) infection remain poorly defined. Here we show that early growth response 1 (EGR1) is independently regulated by each of the three classical MAPK branches including extracellular signal-regulated kinase (ERK), p38 and c-Jun N-terminal kinase (JNK), and serves as a common downstream target of these pathways to promote EV71 replication and virus-induced cell death. Furthermore, we found that the natural isoflavone calycosin (CA) from *Astragalus membranaceus* acts as a regulatory probe of the MAPK-EGR1 axis. CA suppresses EV71-induced expression and phosphorylation of ERK, p38, and JNK, as well as the subsequent upregulation of EGR1, thereby inhibiting both viral replication and virus-induced cell death in conventional cell lines and human colonic organoid infection models. This pro-viral axis is conserved among several enteroviruses, including coxsackievirus B3 and enterovirus D68, and CA exhibits broad-spectrum activity against multiple enteroviruses by targeting this axis. Collectively, our findings establish the MAPK‑EGR1 axis as a key host‑dependency node for EV71 and other enteroviruses, and identify CA as a valuable chemical probe for interrogating this axis. This work not only deepens our understanding of the interplay between enteroviruses such as EV71 and their host, but also provides a mechanistic basis for host‑directed antiviral strategies.

## Introduction

Human enteroviruses (EVs), including echoviruses, coxsackieviruses and numbered enteroviruses such as enterovirus 71 (EV71) and enterovirus D68 (EVD68), belong to the *Picornaviridae* family and can cause a wide spectrum of diseases, ranging from hand, foot and mouth disease (HFMD) to encephalitis, myocarditis, acute flaccid myelitis (AFM), pneumonia, and bronchiolitis. EV71 is one of the most prevalent pathogens responsible for severe HFMD in children and has therefore attracted extensive research attention [1].

Upon infection, viruses often manipulate host factors and signaling pathways to reshape the cellular microenvironment for their efficient replication and survival [2]. In the case of EV71, infection can induce various forms of programmed cell death (PCD), including apoptosis, necroptosis, and pyroptosis. These different forms of cell death not only facilitate viral progeny release but are also implicated in the tissue damage and pathological processes induced by the virus [3,4]. The mitogen‑activated protein kinase (MAPK) cascades, which are central regulators of cell proliferation, differentiation, and apoptosis, are critically involved in EV71 infection [5]. The three canonical MAPK subgroups including extracellular signal-regulated kinase (ERK), p38, and c-Jun N-terminal kinase (JNK) are rapidly activated following EV71 infection, and blockade of these pathways severely suppresses EV71 replication and virus-induced inflammation and apoptosis [6–13]. Similar MAPK-dependent pro-viral mechanisms have also been documented in coxsackievirus B3 (CVB3) infection [14–16]. Nevertheless, the specific downstream effectors that mediate the pro-viral functions of the three MAPK pathways during enterovirus infection remain poorly defined.

Early growth response 1 (EGR1) is a zinc-finger transcription factor that participates in multiple physiological and pathological processes, including tumorigenesis, cardiovascular disorders, inflammatory diseases, and viral infections. Accumulating studies have demonstrated dual roles of EGR1 in viral pathogenesis, acting as either an antiviral or pro-viral regulator in a virus-dependent manner [17,18]. During EV71 infection, EGR1 enhances viral replication by directly binding to the 5′ untranslated region of the EV71 genome or by modulating microRNA-141 gene expression [19,20]. Notably, EGR1 transcription can be independently triggered by all three canonical MAPK pathways. Specifically, ERK-mediated Elk-1 phosphorylation, JNK-induced c-Jun activation, and p38-dependent CREB signaling collectively promote EGR1 promoter activity, indicating that the three MAPK cascades converge at the EGR1 transcriptional level [21–23]. These observations imply that EGR1 may serve as a core downstream effector of MAPK signaling during EV71 infection. If so, targeting the MAPK-EGR1 axis may represent a feasible antiviral strategy.

Calycosin (CA), a natural isoflavone isolated from *Astragalus membranaceus*, exhibits multiple pharmacological activities, including anti-tumor, anti-inflammatory, and antioxidant effects, partly through regulating NF-κB, MAPK and EGR1 pathways [24–27]. Notably, CA also possesses antiviral activity. It can inhibit porcine reproductive and respiratory syndrome virus replication [28]. Earlier studies also reported its antiviral effect against HIV and CVB3 [29,30]; however, the mechanism underlying its anti-CVB3 activity remains unclear. Our previous study revealed that CA suppresses lytic replication of Kaposi’s sarcoma associated herpesvirus (KSHV) by downregulating EGR1 [31]. Given the pro-viral role of EGR1 in EV71 infection and the regulatory effect of CA on EGR1 expression [19,27,31], we hypothesized that CA may exert anti-EV71 activity by targeting the MAPK-EGR1 axis.

Human colonic organoids (HCOs) faithfully recapitulate the physiological characteristics of the human intestinal epithelium and have emerged as a reliable physiological model for investigating enteric viral infections, compensating for the limitations of conventional cell lines [32–34]. In the present study, we combined traditional cell models and HCO systems, using CA as a molecular probe, to explore the functional role of the MAPK-EGR1 axis in EV71 infection and to determine whether this pro-viral mechanism is conserved among other enteroviruses. Our findings provide novel mechanistic insights into enterovirus-host interactions and identify a promising host-targeted pathway for future antiviral development.

## Materials and methods

### Chemicals and reagents

CA (purity > 98%) was purchased from Baoji Herbest Bio-Tech Co., Ltd. (Baoji, Shaanxi, China). Ribavirin (Rb, purity: 99.89%) and SCH772984 (purity: 99.48%) were obtained from Selleck Chemicals LLC (Houston, TX, USA). SB203580 (purity: 99.91%) and SP600125 (purity: 99.46%) were sourced from MedChemExpress (Monmouth Junction, NJ, USA).

### Cells and human colonic organoids

Human rhabdomyosarcoma (RD) cells and African green monkey kidney (Vero) cells were kindly provided by Professor Chao Zhang (Shanghai Institute of Major Infectious Diseases and Biosafety, Fudan university). HeLa and HEK293T cells were purchased from Zhejiang Meisen Cell Technology Co., Ltd (Hangzhou, Zhejiang, China). All cell lines were maintained in DMEM supplemented with 10% fetal bovine serum and 1% penicillin-streptomycin at 37°C in a humidified atmosphere containing 5% CO_2_. Human colonic organoids (HCOs) derived from adult stem cells were provided by Dr. Chao Ni (Nanchang University) and cultured using the Human Colonic Organoid Kit (K2003-HC, bioGenous, Dezhou, China) according to the manufacturer’s instructions. HCOs were passaged weekly, and the medium was refreshed every 3 days.

### Virus strains and titration

EV71 (G082), CA16 (SZ05), CA10 (S0148b), CVB3 (Nancy), EVD68 (US/MO/14–18947), and CA6 (TW-00141) were kindly provided by Professor Chao Zhang (Shanghai Institute of Major Infectious Diseases and Biosafety, Fudan university) and Professor Tong Cheng (Xiamen University). Except for CVB3, which was amplified in HeLa cells, all viruses were propagated in RD cells as described previously [35]. Virus titers were determined via 50% tissue culture infectious dose (TCID_50_) assay using the Reed-Muench method [36].

### Gene overexpression or knockdown

Overexpression vectors, including pLV3-CMV-MAPK14-3×FLAG-mCherry-Puro (P62019), pLV3-CMV-MAPK3-3×FLAG-mCherry-Puro (P87351), and pLV3-CMV-MAPK8-3×FLAG-mCherry-Puro (P87380), as well as the corresponding empty vector pLV3-CMV-MCS-3×FLAG-mCherry-Puro (P54725), were purchased from Wuhan Miaoling Biotechnology Co., Ltd. The EGR1 overexpression vector pLV3-CMV-EGR1 was used as described previously [31]. pLKO.1-U6-EGR1(human)-3UTR-shRNA1-puro targeting EGR1 (P83550) and scrambled control shNC (P0258) were also obtained from the same company. Lentiviruses were produced by co-transfecting HEK293T cells with the overexpression or shRNA vectors, together with the packaging plasmid psPAX2 and envelope plasmid pMD2.G, using polyethyleneimine. RD or HeLa cells were transduced and selected with puromycin to establish stable cell lines with overexpression or knockdown of the respective genes.

### Network pharmacology analysis

Potential targets of CA were retrieved from three public databases: TCMSP (https://www.91tcmsp.com, accessed on 14 May 2025), HERB (http://herb.ac.cn, accessed on 14 May 2025), and INPUT 2.0 (https://cbcb.cdutcm.edu.cn/INPUT/Home, accessed on 14 May 2025). The keyword “calycosin” was used, and all retrieved targets were included without additional confidence filtering. Duplicate targets were removed using Jvenn (https://jvenn.toulouse.inra.fr). All target names were standardized to official gene symbols according to the NCBI Gene database. EV71‑related targets were obtained from GeneCards (https://www.genecards.org, accessed on 4 April 2024), NCBI (https://www.ncbi.nlm.nih.gov/gene, accessed on 8 June 2025), and the GEO dataset GSE103308. For GSE103308, differentially expressed genes were identified using DESeq2 (version 1.12.4) with thresholds of absolute log_2_ fold change > 1.0 and adjusted *p* value ≤ 0.05; genes with very low read counts (baseMean < 10) were excluded. The three source lists were merged, and duplicates were removed using Jvenn. Common targets of CA and EV71 were identified via Venn diagram (Jvenn). Protein‑protein interaction (PPI) network was constructed using the STRING database (https://cn.string-db.org, version 12.0) with the following parameters: organism = “Homo sapiens,” minimum required interaction score = 0.400, and disconnected nodes were hidden. The network was visualized with Cytoscape (version 3.9.0). To identify hub genes without manual bias, six topological algorithms (Degree, MCC, Betweenness, Closeness, Stress, and ClusteringCoefficient) from the cytoHubba plugin were applied. The top 20 targets from each algorithm are provided in Supplementary Table S1. These six lists were intersected using Jvenn to obtain the hub genes, and the resulting subnetwork was visualized with Cytoscape.

## Cytotoxicity and cytoprotective activity assays

### Cell-level assays

RD, Vero, or HeLa cells were seeded in 96-well plates overnight. At approximately 90% confluence, cells were treated with serially diluted test compounds for 72 h (cytotoxicity assay). For cytoprotection at MOI = 0.1, cells were co-treated with CA and EV71 for 2 h, then washed and cultured with fresh CA-containing medium for an additional 70 h. For cytoprotection at MOI = 1, cells were infected with EV71 for 2 h, washed, and then incubated with CA-containing medium for 22 h. Cell viability was assessed using the CCK-8 assay (New cell & Molecular Biotech Co., Ltd, Suzhou, China). For cytotoxicity, viability (%) = [(OD_450_ of experimental wells − OD_450_ of medium control) / (OD_450_ of cell control − OD_450_ of medium control)] × 100%. For cytoprotection, viability (%) = [(OD_450_ of experimental wells − OD_450_ of virus-infected wells) / (OD_450_ of cell control wells − OD_450_ of virus-infected wells)] × 100%. The half-maximal cytotoxic concentration (CC_50_) and half-maximal effective concentration (EC_50_) were derived from dose – response curves using GraphPad Prism. For other cell viability assays (e.g. evaluating the effect of EGR1 knockdown or overexpression on EV71‑induced cell death), the same formula as for cytotoxicity was used, with the cell control representing uninfected cells of the same genetic background (e.g. shNC or empty vector).

### Organoid-level assays

HCOs were treated with serially diluted test compounds for 72 h to assess cytotoxicity. Cell viability was measured using the CellTiter-Glo® 3D Cell Viability Assay kit (Promega, Madison, WI, USA). Briefly, an equal volume of CellTiter-Glo® 3D Reagent was added, and luminescence was read after a 30 min incubation. Viability (%) = (luminescence of drug-treated group / luminescence of DMSO control group) × 100%. For cytoprotection, HCOs were mechanically sheared by pipetting, incubated with diluted virus at 37°C for 2 h, and then centrifuged to remove free virus. Infected HCOs were seeded (1 × 10^3^ cells/well) in 48-well plates pre-coated with 20% Matrigel-organoid growth medium containing test compounds for 70 h. Cell viability was detected using the same kit, and viability (%) = (luminescence of drug-treated group − luminescence of virus-treated group) / (luminescence of DMSO control group − luminescence of virus-treated group) × 100%. CC_50_ and EC_50_ values were calculated as described above.

### Antiviral assays

RD cells, Vero cells, HeLa cells, or HCOs were infected with the indicated virus at an MOI of 1 for 2 h at 37°C as described above. After removal of unbound virus and washing with PBS, the cells were incubated with fresh medium containing serial dilutions of the test compounds. For antiviral activity assessment, virus-infected cells and culture supernatants were collected at 24 h post-infection for Western blot, quantitative reverse transcription‒polymerase chain reaction (RT-qPCR), and virus titer determination. The half-maximal inhibitory concentration (IC_50_) of each compound was calculated by non-linear regression analysis of the dose – response inhibition curve for viral RNA or protein levels using GraphPad Prism.

### Entry and post-entry assays

The entry and post-entry assays were performed in RD cells as described previously [37]. For the post-entry assay, RD cells were infected with EV71 at an MOI of 0.1 for 2 h at 37°C, after which CA was added and the cells were cultured for an additional 20 h. For the entry assay, RD cells were pre-treated with CA for 2 h at 37°C prior to EV71 infection. After 2 h of viral infection, the cells were further cultured for 20 h. EV71 mRNA and VP1 protein levels were then measured by RT-qPCR and Western blot, respectively.

### Western blot analysis

Protein samples were prepared using radioimmunoprecipitation assay buffer (Beyotime, Shanghai, China). After separation by sodium dodecyl sulfate-polyacrylamide gel electrophoresis, the proteins were transferred onto polyvinylidene fluoride membranes. Membranes were blocked with NcmBlot blocking buffer (New cell & Molecular Biotech Co., Ltd), incubated with primary antibodies at 4°C overnight, then washed and incubated with horseradish peroxidase (HRP)-conjugated secondary antibody (1:5000, Abmart, Shanghai, China) at room temperature for 1 h. Positive bands were detected using enhanced chemiluminescence reagents (New cell & Molecular Biotech Co., Ltd). The following primary antibodies were purchased from Proteintech (Wuhan, Hubei, China): rabbit anti-EGR1 antibody (22008–1-AP, 1:1000), mouse anti-GAPDH antibody (HRP6004, 1:1000), rabbit anti-p38 MAPK antibody (80821–3-RR, 1:1000), rabbit anti-Phospho-p38 MAPK (Thr180/Tyr182) antibody (28796–1-AP, 1:1000), rabbit anti-ERK1/2 antibody (83533–1-RR, 1:1000), rabbit anti-JNK antibody (81629–1-RR, 1:1000), rabbit anti-cleaved caspase-3 antibody (25128–1-AP, 1:1000), and mouse anti-PARP antibody (66520–1-Ig, 1:5000). Mouse anti-FLAG Tag antibody (M20038F, 1:1000), rabbit anti-Phospho-ERK1 (T202/Y204) + ERK2 (T185/Y187) antibody (T40072F, 1:1000) and rabbit anti-Phospho-JNK1/2/3 (Thr183/Tyr185) antibody (T40074F, 1:1000) were from Abmart (Shanghai, China); Rabbit anti-EV71 VP1 antibody (1:1000; in-house) and mouse anti-EVD68 VP1 antibody (1:1000; in-house) were prepared and kindly gifted by Professor Chao Zhang (Shanghai Institute of Major Infectious Diseases and Biosafety).

### Immunofluorescence staining

Cells or organoids were fixed with 4% paraformaldehyde at 4°C for 1 h, followed by permeabilization with 0.25% Triton X-100 at room temperature for 30 min. After washing with PBS containing 0.1% Tween-20 (PBST), samples were blocked with 5% bovine serum albumin at room temperature for 1 h, then incubated with primary antibodies at 4°C overnight. Subsequently, samples were washed and incubated with fluorochrome-conjugated secondary antibodies at room temperature for 1 h. Nuclei were stained with 4,’6-diamidino-2-phenylindole (DAPI). Images were captured using a laser scanning confocal microscope (Zeiss, Germany). The primary antibodies used were: rabbit anti-dsRNA antibody (85780–2-RR, Proteintech, 1:200) and mouse anti-E-cadherin monoclonal antibody (60335–1-Ig, Proteintech, 1:200). The secondary antibodies used were: Multi-rAb^TM^ CoraLite® Plus 488 Goat Anti-Rabbit IgG(H+L) (RGAR002, Proteintech, 1:500) and Multi-rAb^TM^ CoraLite® Plus 555 Goat Anti-Mouse IgG(H+L) (RGAM003, Proteintech, 1:500).

### RT-qPCR

Total RNA was extracted using TRIzol reagent (Invitroge^TM^, USA). Complementary DNA was synthesized with HiScript III RT SuperMix kit (R323-01, Vazyme, Nanjing, Jiangsu, China). Quantitative PCR was performed using 2×Realstar Fast SYBR qPCR Mixture (A303, Genstar, Beijing, China). Glyceraldehyde-3-phosphate dehydrogenase (*GAPDH*) was used as the internal reference gene, and the relative expression of each gene was calculated using the 2^− ΔΔCt^ method. All primers are listed in Table S2.

### Statistical analysis

Data are presented as the mean ± standard deviation and analyzed using GraphPad Prism 9.5.1. Group comparisons were performed using paired *t*-tests and one-way analysis of variance, as previously described [31]. A value of *p* < 0.05 was considered statistically significant. For RT‑qPCR data, the y‑axis represents log_2_‑transformed relative expression, and statistical significance is indicated only for changes greater than 2‑fold.

## Results

### EGR1 is a downstream effector of the MAPK pathway and promotes both EV71 replication and virus-induced cell death

**Figure 1.**
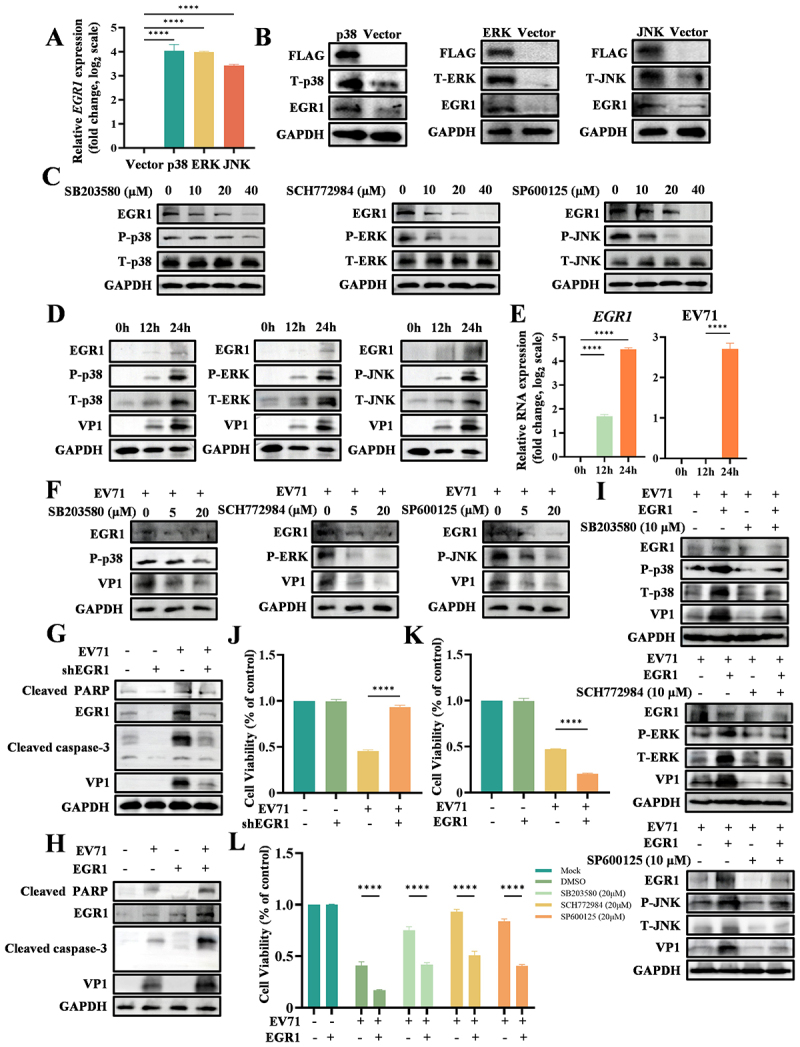
EGR1 promotes EV71 replication and virus-induced cell death by acting as a MAPK downstream effector. (A, B) EGR1 mRNA (A) and protein (B) levels were measured by RT-qPCR and Western blot in RD cells overexpressing p38, ERK, or JNK, respectively. (C) RD cells were treated with the p38 inhibitor SB203580, the ERK inhibitor SCH772984, or the JNK inhibitor SP600125 for 24 h, and EGR1 protein level was assessed by Western blot. (D, E) RD cells were infected with EV71 at an MOI of 1 for the indicated times. Protein levels were analyzed by Western blot (D) and EV71 RNA was tested by RT-qPCR (E). (F) EV71-infected RD cells (MOI = 1) were treated with MAPK inhibitors for 24 h, and the expression of the indicated proteins was examined by Western blot. (G, H, J, K) RD cells transduced with shEGR1 (G, J) or overexpressing EGR1 (H, K) were infected with EV71 (MOI = 1) for 24 h. Protein expression was analyzed by Western blot (G, H), and cell viability was measured by CCK-8 assay (J, K). (I, L) RD cells overexpressing EGR1 were infected with EV71 (MOI = 1) and treated with MAPK inhibitors for 24 h. Protein expression was assessed by Western blot (I), and cell viability was measured by CCK-8 assay (L). GAPDH served as an internal control. Data are presented as means ± standard deviations (*n* = 3 biological replicates). ****p < 0.0001.

To determine whether EGR1 lies downstream of MAPK during EV71 infection, we first overexpressed p38, ERK, or JNK in RD cells. Overexpression of any individual MAPK component significantly upregulated EGR1 mRNA and protein levels (Figure 1(A,B)). Conversely, treatment with the p38 inhibitor SB203580, the ERK inhibitor SCH772984, or the JNK inhibitor SP600125 markedly reduced EGR1 expression (Figure 1(C)), and this reduction was not attributable to cytotoxicity (Figure S1A-C), indicating that EGR1 is a downstream target of MAPK. Furthermore, during EV71 infection, viral replication and the total and phosphorylated levels of p38, ERK, and JNK increased in a time-dependent manner, and EGR1 expression showed a similar trend (Figure 1(D,E)). Treatment with the respective MAPK inhibitors significantly suppressed EV71-induced EGR1 expression and also decreased VP1 protein levels (Figure 1(F)). These data not only confirm that MAPK promotes EV71 replication, but also suggest that EGR1 serves as a key mediator of the pro-viral effect of MAPK.

We next examined the functional role of EGR1 in EV71 replication. Knockdown of EGR1 by specific shRNA significantly reduced EV71 RNA and VP1 protein levels, whereas overexpression of EGR1 enhanced viral replication (Figure 1(G,H); Figure S1D, E). These results are consistent with previous reports that EGR1 acts as a positive regulator of EV71 replication [19]. To further test whether MAPK-promoted EV71 replication depends on EGR1, we overexpressed EGR1 in cells treated with the p38 inhibitor. EGR1 overexpression partially abrogated the antiviral effect of the inhibitor; that is, it restored EV71 replication that had been suppressed by p38 inhibition. Similar results were obtained with the ERK inhibitor SCH772984 and the JNK inhibitor SP600125 (Figure 1(I)). Collectively, these findings indicate that EV71 infection induces MAPK activation, which in turn upregulates EGR1 to enhance viral replication.

Given that MAPK activation contributes to EV71-induced cell apoptosis [11], we tested whether EGR1 mediates this effect. CCK-8 assays showed that EV71 infection caused a marked increase in cell death, and EGR1 knockdown significantly reduced EV71-mediated cell death (Figure 1(J,K)). Moreover, analysis of cleaved PARP and cleaved caspase-3 indicated that EGR1 promotes EV71-induced apoptosis (Figure 1(G,H)). Furthermore, each of the MAPK inhibitors (SB203580, SCH772984, and SP600125) significantly reduced EV71-induced cell death, and this protective effect was reversed by EGR1 overexpression (Figure 1(L)). Collectively, these results demonstrate that EGR1 is a critical downstream effector of the MAPK pathway, promoting both EV71 replication and virus‑induced cell death.

### Network pharmacology predicts the MAPK pathway as a potential target of CA

**Figure 2.**
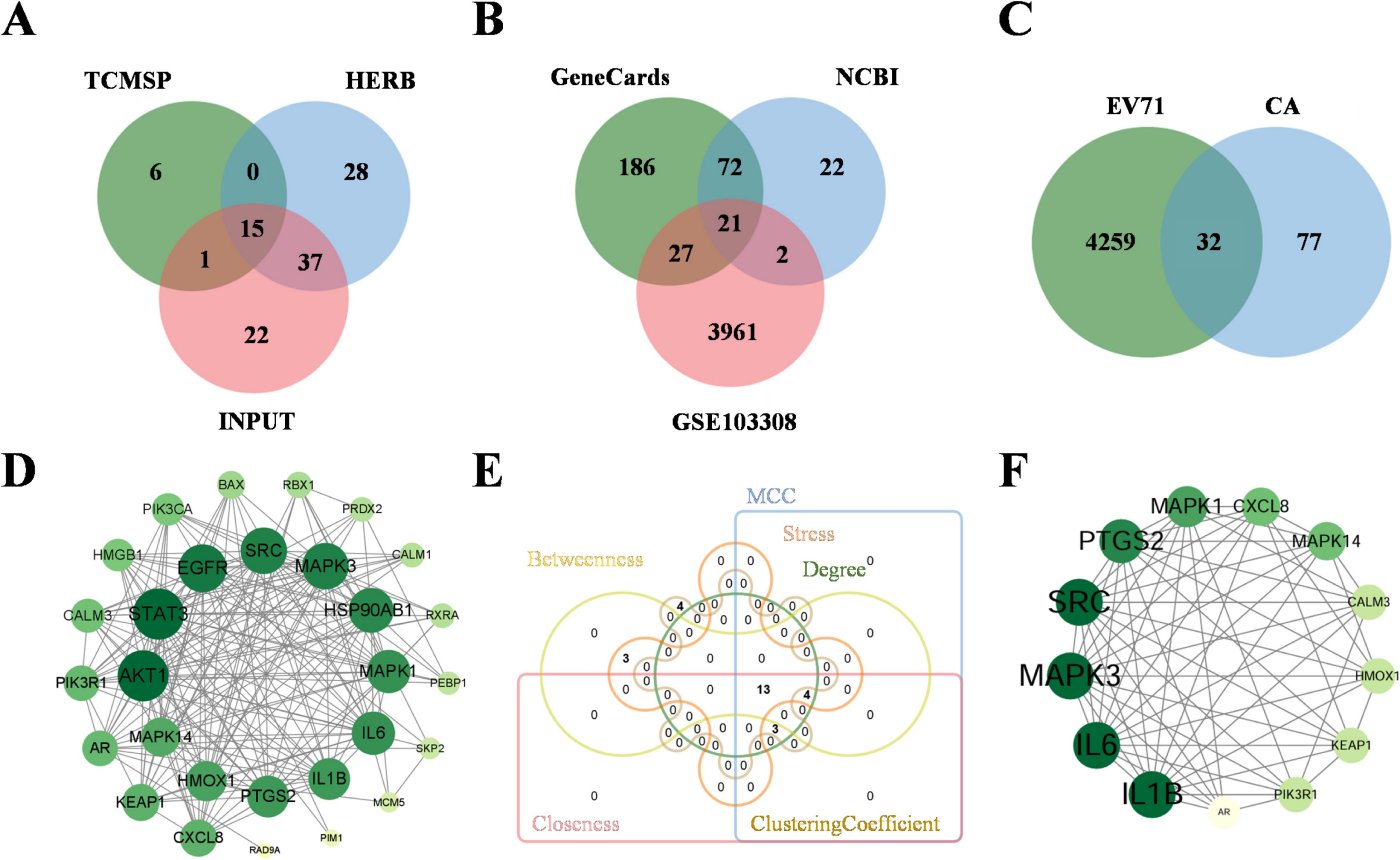
Network pharmacology analysis of calycosin (CA) and EV71 targets. (A) Venn diagram of CA-related targets. (B) Venn diagram of EV71-associated targets. (C) Venn diagram of the common targets between CA and EV71. (D) The protein-protein interaction (PPI) network of the common targets. (E) Venn diagram showing the 13 hub genes identified by intersecting the top 20 targets from six algorithms. (F) The PPI network of the 13 hub genes.

Having established the importance of the MAPK-EGR1 axis and given that CA can modulate MAPK signaling and EGR1 in other contexts [26,31], we next asked whether CA might interfere with this axis during EV71 infection. To test this, we performed a network pharmacology analysis to predict potential CA targets relevant to EV71 infection. CA targets were retrieved from the TCMSP, HERB, and INPUT 2.0 databases, yielding 109 non-redundant targets (Figure 2(A)). EV71-associated targets were obtained from GeneCards, NCBI, and the GEO dataset GSE103308, yielding 4,291 non-redundant targets (Figure 2(B)). Venn diagram analysis identified 32 common targets (Figure 2(C)). The protein-protein interaction (PPI) network of these 32 common targets contained 29 nodes and 195 edges (Figure 2(D)). To systematically identify hub genes without manual bias, we applied six topological algorithms (Degree, MCC, Betweenness, Closeness, Stress, and ClusteringCoefficient) using the cytoHubba plugin (Figure 2(E)). The intersection of the top 20 genes from each algorithm yielded 13 hub genes (Figure 2(F); Table S1). Notably, this hub gene set included three MAPK family members, MAPK3 (ERK1), MAPK1 (ERK2), and MAPK14 (p38). These results imply that CA may regulate EV71 infection by targeting the MAPK pathway.

### CA inhibits EV71 replication and protects against virus-induced cell death

**Figure 3.**
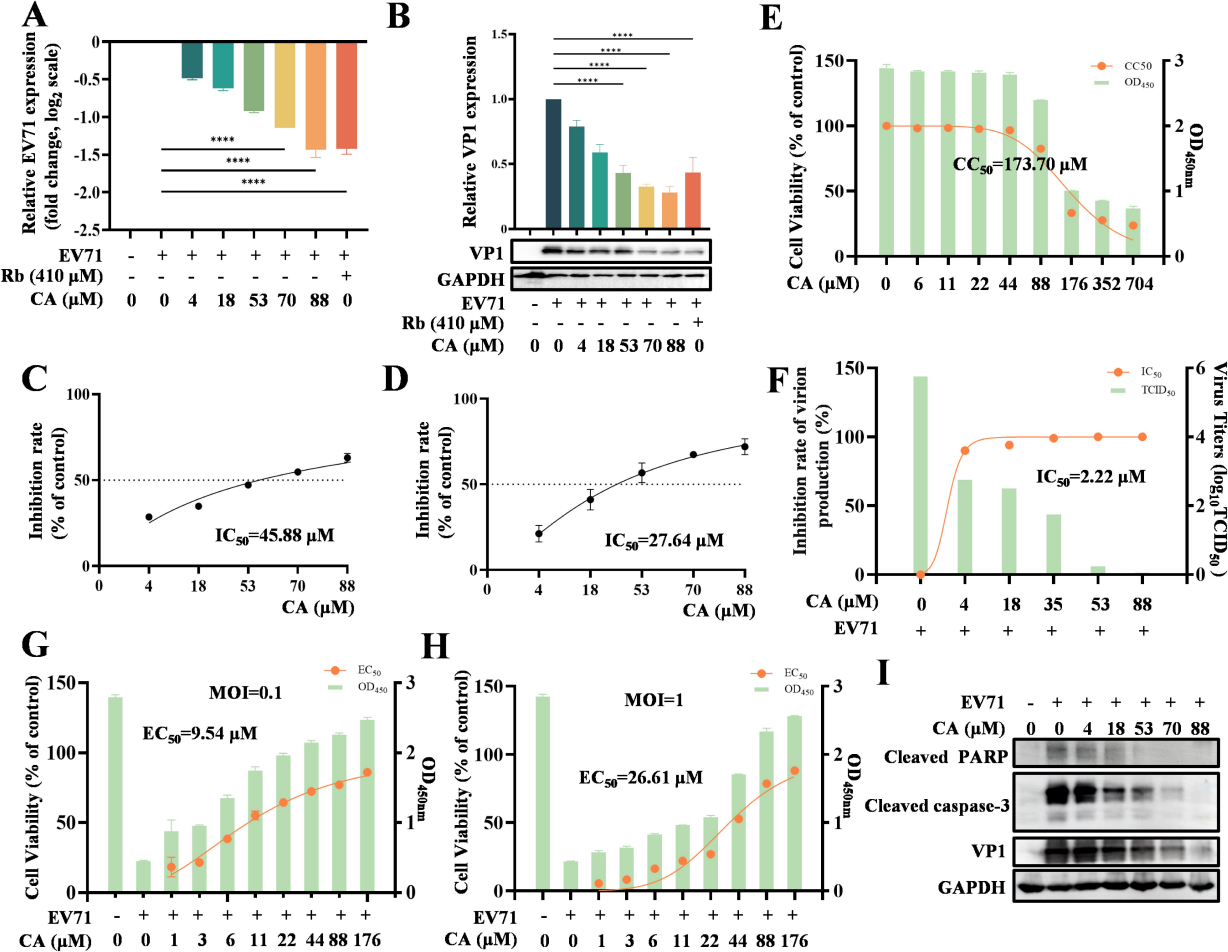
Calycosin (CA) inhibits enterovirus 71 (EV71) replication and virus-induced cell death. (A, B) RD cells were infected with EV71 at MOI = 1 for 2 h and then incubated with increasing concentrations of CA for 22 h. EV71 RNA levels were measured by RT-qPCR (A), and VP1 protein levels were detected by Western blot (B). (C, D) half-maximal inhibitory concentration (IC_50_) curves for viral RNA (C) and VP1 protein (D) derived from the data in (A) and (B), respectively. (E) cytotoxicity of CA in RD cells; the half-maximal cytotoxic concentration (CC_50_) curve is presented. (F) progeny virus titers (TCID_50_) in supernatants from the cells described in (A); the IC_50_ curve was determined. (G) RD cells were infected with EV71 at MOI = 0.1 and treated with various concentrations of CA for 72 h. Cell viability was measured by CCK-8 assay, and the half-maximal effective concentration (EC_50_) curve was calculated. (H, I) RD cells were infected with EV71 at MOI = 1 for 2 h and then treated with increasing concentrations of CA for 22 h. Cell viability was measured by CCK-8 assay, and the EC_50_ curve was generated (H); Western blot analysis of the indicated proteins is shown (I). Data are presented as mean ± standard deviations (*n* = 3 biological replicates). ****p < 0.0001.

To validate the network pharmacology prediction, the antiviral activity of CA against EV71 was first examined, using ribavirin as a positive control (Figure S2A-C). In RD cells, CA dose-dependently reduced EV71 RNA and VP1 protein levels, with IC_50_ values of 45.88 μM and 27.64 μM, respectively (Figure 3(A–D)), and showed no obvious cytotoxicity (CC_50_ = 173.70 μM, Figure 3(E)). Under the same infection conditions (MOI = 1, 24 h), CA also significantly inhibited progeny virus production (TCID_50_) with an IC_50_ of 2.22 μM (Figure 3(F)). Next, we examined the cytoprotective effect of CA. Under the initial condition (MOI = 0.1, 72 h), CA dose-dependently increased cell viability with an EC_50_ of 9.54 μM (Figure 3(G)). To directly compare the antiviral and cytoprotective activities of CA under identical infection conditions, the EC_50_ for cytoprotection at MOI = 1, 24 h was also determined. This value was 26.61 μM (Figure 3(H)), which is comparable to the antiviral IC_50_ values for VP1 protein. These results indicate that under matched infection conditions, CA suppresses both EV71 replication and cell death with similar potency. To further determine whether the cytoprotective effect of CA is attributable to inhibition of apoptosis, Western blot analysis was performed, showing that CA reduced cleaved PARP and cleaved caspase-3 levels, indicating that CA suppresses EV71-induced apoptosis (Figure 3(I)). Similar results were observed in Vero cells (Figure S2D-J).

The stage at which CA acts was then determined. As shown in Figure S3, CA effectively inhibited viral replication and cell death when added after viral entry, but not during the entry step, indicating that CA acts primarily at the post-entry stage.

### CA suppresses EV71-induced MAPK expression

**Figure 4.**
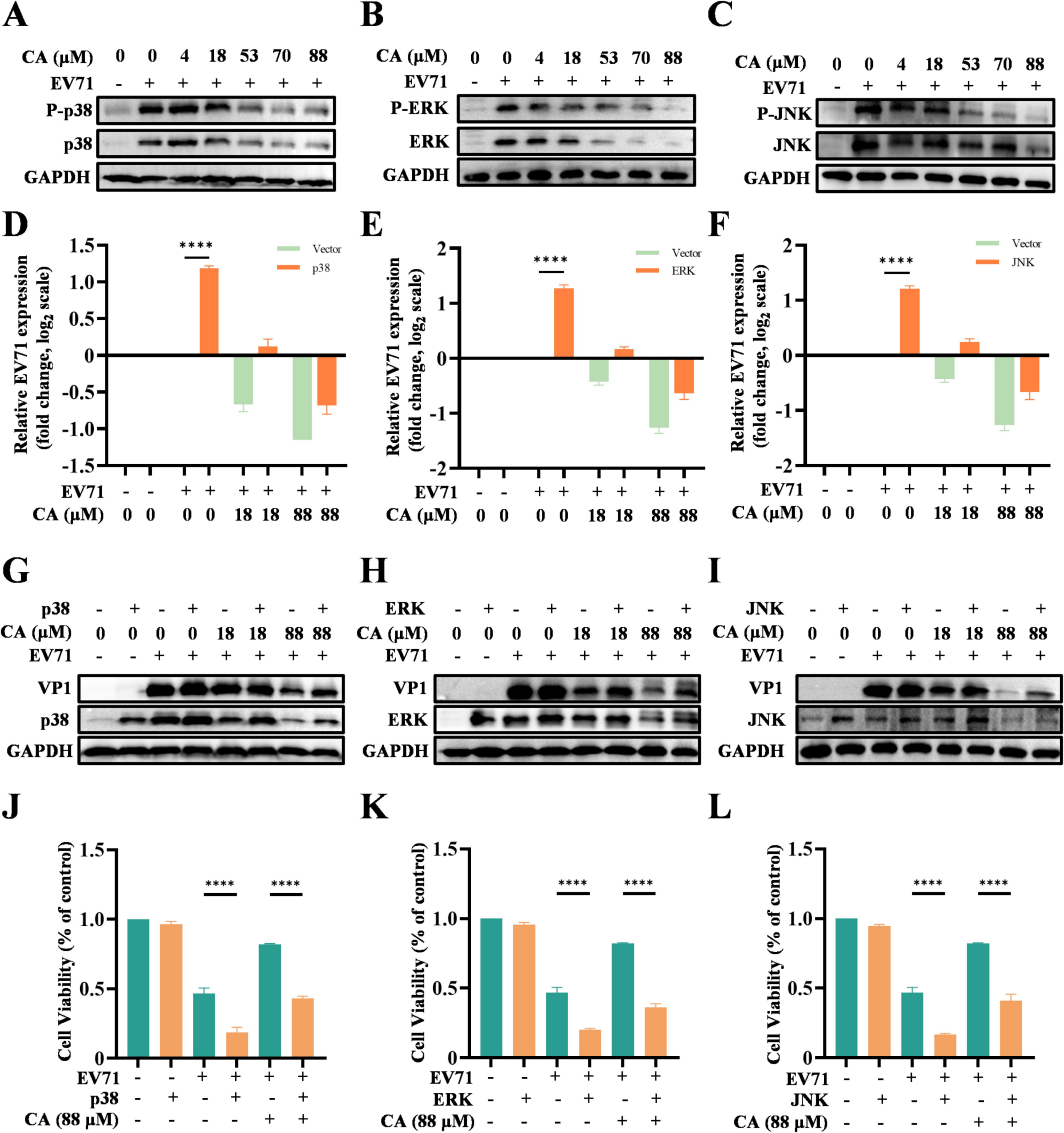
The mitogen-activated protein kinase (MAPK) molecules are involved in the antiviral effect of calycosin (CA). (A-C) RD cells were infected with EV71 (MOI = 1, 2 h) and then treated with increasing concentrations of CA for 22 h. Western blots show the total and phosphorylated p38 (A), ERK (B), and JNK (C). (D-L) RD cells overexpressing p38, ERK, or JNK were infected with EV71 at MOI = 1 for 2 h and then treated with CA for 22 h. Viral RNA levels were measured by RT-qPCR (D-F); VP1 protein levels were detected by Western blot (G-I); cell viability was measured by CCK-8 assay (J-L). These panels correspond to p38, ERK, and JNK overexpression, respectively. Data are presented as mean ± standard deviations (*n* = 3 biological replicates). ****p < 0.0001.

To confirm that the MAPK molecules predicted by network pharmacology are targeted by CA, we examined the effect of CA on p38 and ERK, and additionally on JNK, all of which are essential for EV71 replication. As expected, CA treatment dose-dependently reduced both the total protein levels and the phosphorylation of these MAPKs following EV71 infection (Figure 4(A–C)), without affecting their basal expression in uninfected cells (Figure S4A), indicating selective inhibition during EV71 infection. Rescue experiments further verified that the antiviral and cytoprotective effects of CA depend on MAPK inhibition. Overexpression of each of these MAPKs (p38, ERK, or JNK) in EV71-infected RD cells significantly increased viral RNA and VP1 levels and partially restored viral replication in the presence of CA (Figure 4(D–I)). Moreover, while CA increased cell viability, overexpression of these MAPKs enhanced EV71-induced cell death and reversed CA’s protective effect (Figure 4(J–L)). Collectively, these results indicate that CA suppresses EV71 replication and protects against virus-induced cell death by inhibiting MAPK expression.

### CA acts through the MAPK-EGR1 axis to inhibit EV71 replication and cell death

**Figure 5.**
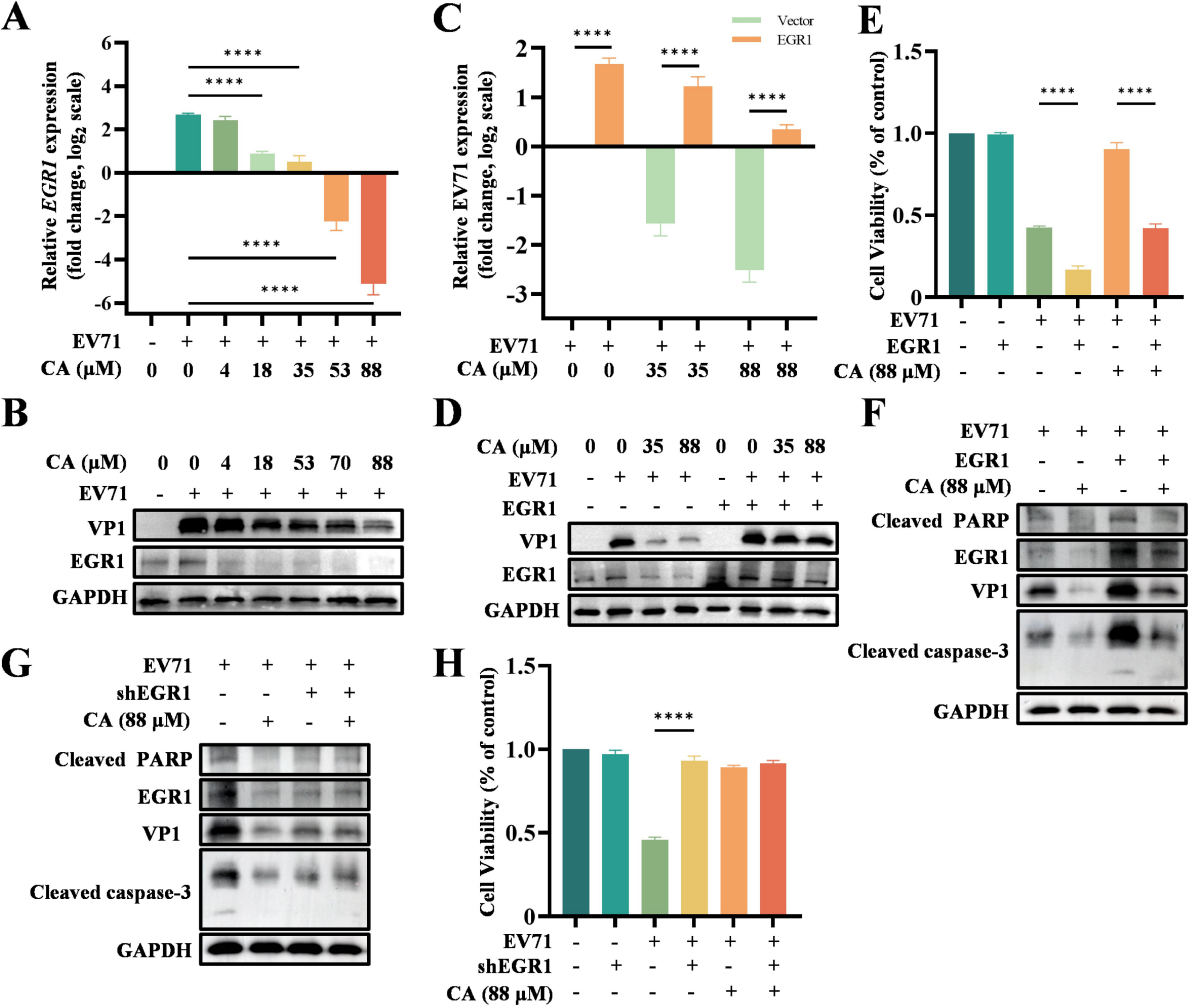
EGR1 is required for calycosin’s antiviral and cytoprotective effects. (A, B) EV71-infected RD cells (MOI = 1, 2 h) were treated with CA for 22 h. EGR1 mRNA (A) and protein (B) levels were measured by RT-qPCR and Western blot. (C, D) EGR1-overexpressing RD cells were infected with EV71 (MOI = 1, 2 h) followed by CA treatment for 22 h. EV71 RNA (C) and VP1 protein (D) levels were assessed. (E, F) under the same conditions, cell viability (E) and apoptotic proteins (cleaved PARP, cleaved caspase-3) (F) were measured in EGR1-overexpressing cells. (G, H) in shEGR1-transduced RD cells, apoptotic proteins (G) and cell viability (H) were similarly analyzed. The error bars represent the standard deviations of 3 independent repeats. ****p < 0.0001.

To further determine whether the MAPK-EGR1 axis is targeted by CA, we first examined the effect of CA on EGR1 expression. Similar to the results obtained with MAPK inhibitors, CA dose-dependently suppressed EV71-induced EGR1 mRNA and protein expression (Figure 5(A,B)). However, CA did not affect the basal level of EGR1 expression (Figure S4A, B). Overexpression of EGR1 significantly reversed the inhibitory effect of CA on viral replication, as evidenced by increased viral RNA and VP1 levels (Figure 5(C,D)). Moreover, the protective effect of CA against cell death was markedly attenuated by EGR1 overexpression (Figure 5(E)), and the virus-induced apoptotic markers suppressed by CA were also significantly restored (Figure 5(F)). Furthermore, in EGR1 knockdown cells (shEGR1), CA treatment failed to further reduce VP1 levels compared to shEGR1 cells without CA, whereas CA significantly inhibited viral replication in control shNC cells (Figure 5(G)). A similar pattern was observed against cell death protection (Figure 5(H)). These results indicate that both the antiviral and cytoprotective effects of CA depend on EGR1. Together with the findings that CA inhibits MAPK expression and that the MAPK-EGR1 axis plays a pro-viral role in EV71 replication, these data demonstrate that CA serves as a regulatory probe targeting the MAPK-EGR1 axis to suppress EV71 replication and protect against virus-induced cell death.

### The conserved MAPK-EGR1 axis mediates the broad-spectrum anti-enterovirus activity of CA

**Figure 6.**
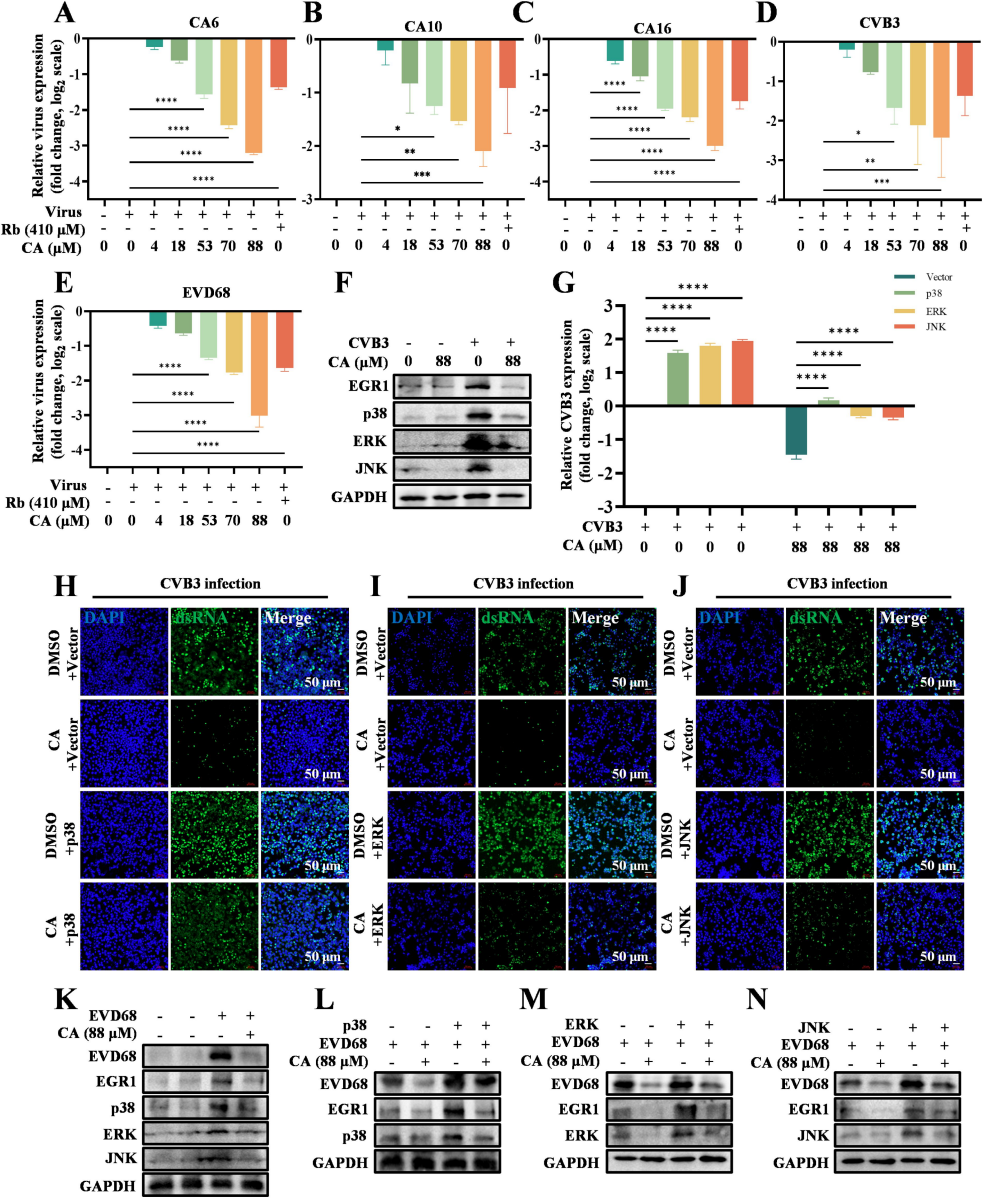
CA exhibits broad-spectrum activity against enteroviruses via the conserved MAPK-EGR1 axis. (A-E) relative viral RNA levels of coxsackievirus A6 (CA6, A), A10 (CA10, B), A16 (CA16, C), coxsackievirus B3 (CVB3, D), and enterovirus D68 (EVD68, E). Cells were infected at MOI = 1 for 2 h, then treated with CA for 22 h. CVB3 was assayed in HeLa cells; others in RD cells. (F, K) Western blot analysis of total p38, ERK, JNK, and EGR1 protein levels in CVB3-infected HeLa cells (F) and EVD68-infected RD cells (K) treated with CA under the same conditions. (G) RT-qPCR quantification of CVB3 RNA in HeLa cells overexpressing p38, ERK, or JNK, with or without CA treatment. (H-J) immunofluorescence detection of CVB3 RNA in HeLa cells overexpressing p38 (H), ERK (I), or JNK (J), with or without CA treatment. (L-N) Western blot analysis of EVD68 VP1, EGR1, and overexpressed p38 (L), ERK (M), or JNK (N) in stably overexpressing RD cells under the same infection and treatment conditions. The error bars represent the standard deviations of 3 independent repeats. *p < 0.05, **p < 0.01, ***p < 0.001, ****p < 0.0001.

To investigate whether the MAPK-EGR1 axis is also conserved in other enteroviruses, we used CA as a regulatory probe to evaluate its broad-spectrum antiviral activity. We first tested the inhibitory effect of CA against several enteroviruses, including coxsackievirus A6 (CA6), A10 (CA10), A16 (CA16), CVB3, and EVD68. CA significantly reduced the RNA levels of all tested viruses (Figure 6(A–E)), with IC_50_ values shown in Figure S5A-E. CCK-8 assays further demonstrated that CA effectively protected cells from cell death induced by these viral infections (Figure S5F-J). Using CVB3 and EVD68 as representatives, we further examined whether the MAPK-EGR1 axis is involved. As observed with EV71, CA suppressed virus-induced MAPK and EGR1 expression in CVB3-infected HeLa cells without cytotoxicity (Figure 6(F), Figure S5K), as well as in EVD68-infected RD cells (Figure 6(K)). Moreover, individual overexpression of p38, ERK, or JNK reversed the inhibitory effect of CA on CVB3 and EVD68 replication (Figure 6(G–J),(L–N)). Importantly, CCK-8 assays showed that CA treatment significantly increased the viability of EVD68-infected cells, and this protective effect was also reversed by overexpression of p38, ERK, or JNK (Figure S5L-N). Together, these results indicate that the conserved MAPK-EGR1 axis mediates the broad-spectrum anti-enterovirus activity of CA, suggesting that this axis plays a conserved pro-viral role across different enteroviruses.

### Regulation of the MAPK‑EGR1 axis by CA during EV71 infection in human colonic organoids

**Figure 7.**
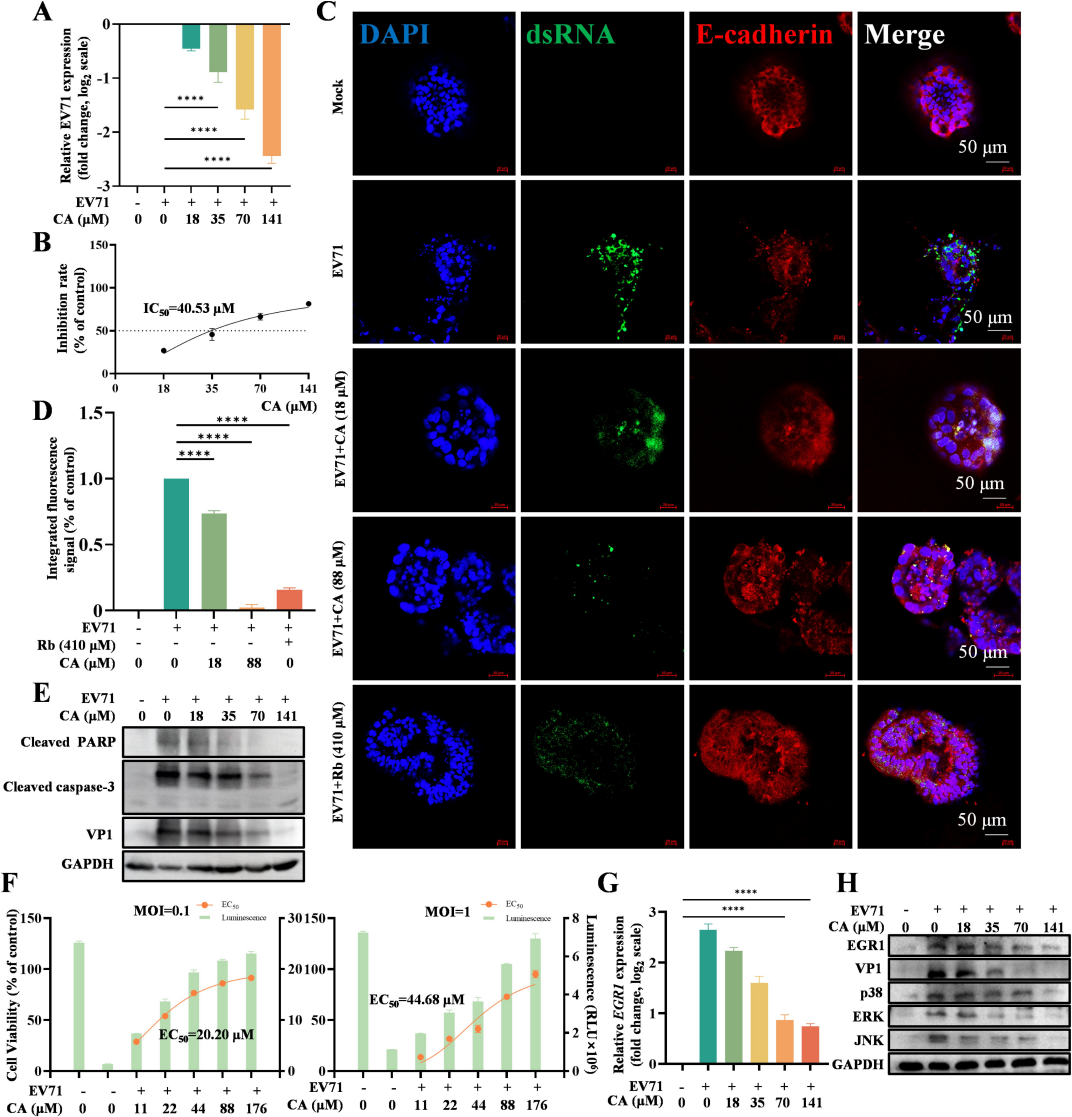
Calycosin (CA) exhibits antiviral effects in human colonic organoids (HCOs). (A, B) HCOs were infected with EV71 (MOI = 1, 2 h) and then treated with CA for 22 h. EV71 RNA levels were measured by RT- qPCR (A), and the half-maximal inhibitory concentration (IC_50)_ curve is shown in (B). (C, D) immunofluorescence staining of viral dsRNA (green), enterocyte marker E-cadherin (red) and DAPI-stained nuclei in HCOs treated as in (A) (C), with statistical analysis of fluorescence intensity shown in (D). (E) Western blot analysis of EV71 VP1 and apoptotic markers in HCOs under the same conditions. (F) HCOs were infected with EV71 at MOI = 0.1 or 1 for 2 h and then treated with CA for 70 h. Cell viability was measured by CellTiter-Glo® 3D cell viability assay kit, and the half-maximal effective concentration (EC_50_) curves are shown. (G, H) under the same HCO infection conditions as in (A), EGR1 mRNA levels were assessed by RT-qPCR (G), and protein expressions of EGR1 and other indicated proteins were analyzed by Western blot (H). Data are presented as mean ± standard deviations (*n* = 3 independent repeats). ****p < 0.0001.

To validate the regulation of the MAPK-EGR1 axis by CA in a physiologically relevant human model, we used human colonic organoids (HCOs). EV71 efficiently infected HCOs, leading to obvious cytopathic effects and increased viral RNA levels (Figure S6A, B). As shown in Figure 7(A–E), CA treatment dose-dependently reduced EV71 RNA levels and VP1 protein with concentrations far below the CC_50_ of CA in HCOs (Figure S6C), indicating suppression of viral replication. Ribavirin (Rb) was also used as a positive control (Figure S6D, E). Moreover, CA protected HCOs from EV71-induced cell death (Figure 7(F)) and reduced cleaved PARP and cleaved caspase-3 (Figure 7(E)), confirming inhibition of apoptosis. To directly assess whether CA modulates the MAPK-EGR1 axis, we examined the expression of MAPK components and EGR1. Consistent with cell line data, CA treatment significantly suppressed EV71-induced p38, ERK, JNK, and EGR1 expression (Figure 7(G,H)). These results further confirm that the MAPK-EGR1 axis plays a pro-viral role in EV71 replication and that CA suppresses viral replication and protects against virus induced cell death by targeting this axis in the HCO model.

## Discussion

Many viruses hijack MAPK pathways to modulate their replication, albeit with divergent regulatory effects among different viruses [38,39]. EV71 activates p38, JNK, and ERK to promote viral replication and virus‑induced apoptosis [11,40]. However, the downstream effectors have remained unclear. Here we identify EGR1 as a common downstream target of these MAPK pathways during EV71 infection. Using CA, a natural compound from *Astragalus membranaceus*, as a chemical probe, we further demonstrate that CA suppresses EV71-induced expression and activation of p38, JNK, ERK and downstream EGR1, thereby inhibiting both viral replication and virus-induced cell death. This study reveals the pro-viral function of the MAPK‑EGR1 axis during EV71 infection, and this axis is functionally conserved among several other enteroviruses, such as CVB3 and EVD68. Thus, our findings not only deepen the understanding of enterovirus-host interactions but also provide a new rationale for antiviral strategies targeting this axis.

MAPKs belong to the serine/threonine kinase family and play essential regulatory roles in cellular processes such as proliferation, differentiation, migration, death and stress responses [41]. To date, fourteen MAPK members have been identified in mammalian cells, classified into seven subgroups: extracellular regulated kinases (ERK1/2), p38 MAPK (p38α/β/γ/δ), c-Jun NH2-terminal kinases (JNK1/2/3), ERK3/4, ERK5, ERK7/8, and Nemo-like kinase (NLK). Among them, ERK1/2, p38, and JNK are well-characterized and represent the three classical MAPK signaling cascades [42]. These cascades differ in their upstream activators and downstream targets, leading to distinct functional preferences. p38 and JNK are primarily activated by extracellular stress stimuli such as UV irradiation, heat shock, and inflammatory cytokines, and their activation promotes apoptosis and the expression of pro-inflammatory cytokines. ERK1/2 mainly responds to growth factors, promotes cell proliferation and differentiation, and can also activate anti-apoptotic proteins to help cells evade programmed cell death. However, the specific outcomes of MAPK activation depend strongly on cellular context [8,43].

Nevertheless, these three classical MAPK cascades exhibit consistent pro-viral functions in the context of EV71 infection. For instance, inhibition of ERK1/2 in RD cells markedly reduces EV71 replication [13]. In immature dendritic cells, EV71 infection promotes the expression and phosphorylation of p38 and JNK; inhibition of these molecules decreases viral replication and lowers the levels of virus-induced inflammatory cytokines IL-6, IL-10 and TNF-α [9]. Moreover, a specific p38 inhibitor has been reported to strongly suppress EV71-induced apoptosis [11]. In addition, in CVB3 infection, activation of JNK and p38 occurs during replication, and a p38 inhibitor effectively reduces progeny virus release and virus-induced cell death [16]. Our results confirm these observations and extend them to other enteroviruses, such as CA6, CA10, CA16, and EVD68, in which the same pro-viral mechanisms are conserved. This work thus provides further experimental support for antiviral strategies that target MAPK pathways.

Furthermore, we demonstrated that p38, JNK and ERK each act via the common downstream target EGR1 during EV71 infection. EGR1 is a multifunctional transcription factor that regulates numerous downstream target genes involved in apoptosis, and has been implicated in infections such as HIV, HCV, HSV and KSHV [17]. Here, consistent with previous reports that EGR1 promotes EV71 replication [19], our data further demonstrate that EGR1 also enhances virus-induced cell death. This enhanced cell death may be partly attributable to increased viral replication; however, our data suggest that EGR1 may also exert direct pro-apoptotic effects. Earlier studies have reported that EGR1 upregulates the expression of p53 and p21, both of which are critical regulators of apoptosis [17]. In the context of enterovirus infection, EV71 infection promotes p53 expression [44], and CA6 also induces p53 and p21 upregulation during infection [45]. Although the specific downstream targets of EGR1 that contribute to EV71 pathogenesis remain to be identified, it is possible that EGR1 exerts its pro‑apoptotic effects in part through pathways involving p53 and/or p21. Beyond its role in EV71, EGR1 has also been shown to promote viral replication in other infections. For example, during vaccinia virus infection, MAPK signaling is activated and upregulates EGR1; in various cell lines such as fibroblasts, the MEK/ERK/EGR1 pathway promotes vaccinia virus proliferation, and downregulation of EGR1 markedly reduces viral replication [46]. Given that MAPK pathways are exploited by multiple viruses [39], these observations indicate that the MAPK-EGR1 axis is a conserved pro-viral mechanism across different viruses.

Interestingly, the present study also indicated that CA can act as a regulator of this axis. CA selectively suppresses EV71-induced upregulation of MAPKs and EGR1 without affecting their basal levels, indicating favorable specificity in its anti-EV71 activity. Notably, the IC_50_ of CA for progeny virus production (2.22 μM) was substantially lower than that for viral RNA (45.88 μM) or VP1 protein (27.64 μM). This discrepancy may be attributable to the dual effects of CA, which can both inhibit viral replication and protect against virus-induced cell death. Since the TCID_50_ assay measures progeny virus released into the supernatant, and virus release is closely linked to virus-induced cell death and lysis [47], the observed difference further supports the dual regulatory role of CA. Thus, even though CA exhibits only moderate antiviral potency compared with the potent direct-acting antiviral drug rupintrivir [48], we have successfully used this probe to reveal the critical role of the MAPK-EGR1 axis in enterovirus infection. Therefore, attention to the MAPK-EGR1 axis may open new avenues for understanding the infection mechanisms of diverse viruses, and CA provides a valuable tool for exploring the functions of this axis in other physiological or pathological settings.

This study has two limitations. First, EV71 triggers multiple forms of programmed cell death including apoptosis, necroptosis, and pyroptosis [4,49,50]. We primarily assessed total cell death by CCK-8 assays and evaluated apoptosis by measuring cleaved PARP and cleaved caspase-3 levels. Although these data sufficiently support the core conclusions, we cannot fully exclude the involvement of other cell death pathways. Second, this study focused on dissecting the mechanistic function of the MAPK-EGR1 axis using CA as a chemical probe rather than evaluating its *in vivo* efficacy. Therefore, we did not perform animal infection experiments. Instead, we employed HCOs, which recapitulate human intestinal epithelial physiology and help verify our mechanistic findings in a human-relevant system. Of note, oral CA yields low systemic plasma levels, yet portal vein concentrations can peak at 10.9 μM, with a hepatic first-pass effect as high as 98.5% [51]. Because CA is absorbed in the intestine [25], its local concentration in intestinal epithelial cells is likely even higher than that in the portal vein. Given that EV71 primarily replicates in the intestine, this local enrichment may allow CA to reach effective concentrations at the primary site of infection. Consistently, our HCO experiments showed that CA protected EV71-infected cells with EC_50_ values of 20.20 μM (MOI = 0.1) and 44.68 μM (MOI = 1). Although the higher EC^50^ at MOI = 1 exceeds the portal vein concentration, the local intestinal concentration during absorption may be sufficient to achieve the observed antiviral effects. Relevant animal experiments will be carried out in future work.

## Conclusion

This study demonstrates that the MAPK-EGR1 axis is critical for EV71 replication and virus-induced cell death. Here, CA, used as a chemical probe, specifically dampens EV71-triggered MAPK activation and subsequent EGR1 upregulation, thereby suppressing both viral replication and virus-induced cell death. Moreover, this axis appears to be functionally conserved among several enteroviruses, highlighting its potential as a common host dependency node. Collectively, our findings provide a rationale for targeting this axis in antiviral strategies and establish CA as a useful tool for dissecting MAPK-EGR1 signaling.

## Supplementary Material

Supplemental Material

supplementary table clean 0615.docx

Revised Supplementary file clean.docx

## Data Availability

The raw data was deposited in a recognized data repository (www.Figshare.com) with a digital object identifier (https://doi.org/10.6084/m9.figshare.31305175) [52].
