# Supplementary material for: Calycosin targets the MAPK-EGR1 signaling axis to inhibit enterovirus 71 replication in cells and human colonic organoids
Source: Virulence. 2026 Aug 5;17(1):2711500. doi: 10.1080/21505594.2026.2711500 (PMC13449819; doi:10.1080/21505594.2026.2711500)
Supplement: Supplementary file 1 [file KVIR_A_2711500_SM6807.docx]

**Supplementary Figure 1. The cytotoxicity of MAPK inhibitors and detection of relative RNA levels.** (A-C) Cytotoxicity of the p38 inhibitor SB203580 (A), the ERK inhibitor SCH772984 (B), and the JNK inhibitor SP600125 (C) against RD cells. The half‑maximal cytotoxic concentration (CC₅₀) curve is presented for each inhibitor. (D, E) RD cells transduced with shEGR1 (D) or overexpressing EGR1 (E) were infected with EV71 (MOI=1) for 24 h. EV71 RNA and EGR1 mRNA were measured by RT‑qPCR.

**Supplementary Figure 2. Cytotoxicity and antiviral activity of ribavirin (Rb) and calycosin (CA).** (A) Half-maximal cytotoxic concentration (CC₅₀) of Rb against RD cells. (B) RD cells were infected with EV71 at MOI=0.1 and treated with various concentrations of Rb for 72 h. Cell viability was measured by CCK‑8 assay, and the half‑maximal effective concentration (EC₅₀) curve was fitted. (C) Progeny virus titers (TCID₅₀) in supernatants from RD cells infected with EV71 at MOI=1 for 2 h and then treated with Rb for 22 h. (D) CC₅₀ of CA against Vero cells. (E, G) Vero cells were infected with EV71 at MOI=1 for 2 h and then incubated with increasing concentrations of CA for 22 h. EV71 RNA levels were measured by RT‑qPCR (E), and VP1 protein levels were detected by Western blot (G). (F, H) Half‑maximal inhibitory concentration (IC₅₀) curves for viral RNA (F) and VP1 protein (H) derived from the data in (E) and (G), respectively. (I) Progeny virus titers (TCID₅₀) in supernatants from the cells described in (E); the corresponding IC₅₀ curve is shown. (J) Under the same conditions as in (E, G), Vero cells were treated with CA; cell viability and EC₅₀ curve are shown.

**Supplementary Figure 3. Calycosin (CA) inhibits enterovirus 71 (EV71) replication at the post-entry stage.** (A) Schematic of the EV71 entry assay. (B, C) EV71 RNA (B) and VP1 protein (C) levels in infected RD cells after the entry assay in (A). (D) Schematic of the post-entry assay. (E, F) EV71 RNA (E) and VP1 protein (F) levels in infected RD cells after the post-entry assay in (D).

**Supplementary Figure 4. Calycosin (CA) does not affect the basal levels of MAPK and EGR1.** (A) RD cells were treated with CA for 24 h. Total protein levels of p38, ERK, JNK, and EGR1 were measured by Western blot. (B) EGR1 mRNA levels were assessed by RT‑qPCR from cells treated as in (A).

**Supplementary Figure 5. The broad-spectrum antiviral activity of calycosin (CA).** (A-E) Half‑maximal inhibitory concentration (IC₅₀) curves for viral RNA of coxsackievirus A6 (CA6, A), A10 (CA10, B), A16 (CA16, C), coxsackievirus B3 (CVB3, D), and enterovirus D68 (EVD68, E), derived from the data in Figure 6A-E. (F-J) RD or HeLa cells were infected with indicated virus at MOI=0.1 and treated with various concentrations of CA for 72 h. Cell viability was measured by CCK‑8 assay, and half‑maximal effective concentration (EC₅₀) curves were calculated. (K) Half-maximal cytotoxic concentration (CC₅₀) of CA against HeLa cells. (L-N) RD cells overexpressing p38 (L), ERK (M), or JNK (N) were infected with EVD68 at MOI=1 for 2 h and then treated with CA for 22 h. Cell viability was measured by CCK‑8 assay.

**Supplementary Figure 6. Time course and drug response assays in human colonic organoids (HCOs).** (A) Brightfield images of mock- and EV71-infected HCOs at different time points (scale bar: 50 µm). (B) Relative EV71 RNA levels in infected HCOs at different timepoints. (C, D) Half-maximal cytotoxic concentration (CC₅₀) of CA (C) and Rb (D) against HCOs. (E) HCOs were infected with EV71 at MOI=1 for 2 h and then treated with CA for 70 h. Cell viability was measured by CellTiter‑Glo® 3D Cell Viability Assay kit, and the half‑maximal effective concentration (EC₅₀) curves is shown.
