# Supplementary material for: Calycosin targets the MAPK-EGR1 signaling axis to inhibit enterovirus 71 replication in cells and human colonic organoids
Source: Virulence. 2026 Aug 5;17(1):2711500. doi: 10.1080/21505594.2026.2711500 (PMC13449819; doi:10.1080/21505594.2026.2711500)
Supplement: Supplementary file 2 [file KVIR_A_2711500_SM6808.docx]

**Table S1** The top 20 targets identified by each of the six algorithms.

| **Algorithm** | **Genes** |
| --- | --- |
| **Degree** | *STAT3、AKT1、EGFR、MAPK3、SRC、HSP90AB1、IL6、MAPK1、PTGS2、IL1B、HMOX1、MAPK14、KEAP1、CXCL8、PIK3R1、AR、CALM3、PIK3CA、HMGB1、BAX* |
| **MCC** | *AKT1、STAT3、MAPK3、EGFR、IL6、SRC、IL1B、HSP90AB1、PTGS2、CXCL8、MAPK1、MAPK14、HMOX1、HMGB1、KEAP1、CALM3、AR、PIK3R1、PIK3CA、BAX* |
| **Betweenness** | *STAT3、AR、AKT1、EGFR、HSP90AB1、HMOX1、MAPK1、SRC、MAPK3、PTGS2、MAPK14、IL6、KEAP1、RBX1、IL1B、PIK3R1、SKP2、CXCL8、PRDX2、CALM3* |
| **Closeness** | *STAT3、AKT1、EGFR、MAPK3、SRC、HSP90AB1、IL6、MAPK1、PTGS2、IL1B、HMOX1、MAPK14、KEAP1、CXCL8、AR、PIK3R1、CALM3、PIK3CA、HMGB1、BAX* |
| **Stress** | *STAT3、AKT1、AR、EGFR、HSP90AB1、SRC、MAPK3、MAPK1、HMOX1、IL6、PTGS2、IL1B、MAPK14、KEAP1、PIK3R1、CXCL8、RBX1、SKP2、PRDX2、CALM3* |
| **Clustering Coefficient** | *PIM1、BAX、HMGB1、RXRA、CALM1、PIK3CA、CALM3、CXCL8、IL1B、PEBP1、IL6、KEAP1、PTGS2、PIK3R1、MAPK14、AR、MAPK3、HMOX1、SRC、MAPK1* |

**Table S2** The primers used in this study.

| **Primer name** | **Sequence (5’-3’)** |
| --- | --- |
| GAPDH-F | ATCCCATCACCATCTTCCAGG |
| GAPDH-R | CCTTCTCCATGGTGGTGAAGAC |
| EV71-F | CCCTGAATGCGGCTAATCC |
| EV71-R | ATTGTCACCATAAGCAGCCA |
| p38-F | TGGTGGCACAAAGCTGATGAC |
| p38-R | GCCCAAGCCCTTGCACAT |
| ERK-F | TCATCAACATGAAGGCCCGAA |
| ERK-R | GCTCCTTAGGTAGGTCATCCAG |
| JNK-F | ATGAAGCTCTCCAACACCCG |
| JNK-R | ACTGCTGCACCTGTGCTAAA |
| EGR1-F | CCACGCCGAACACTGACATT |
| EGR1-R | GAGGGGTTAGCGAAGGCTG |
| CA10-F | GGGCTTTGTCCAACTCCGCA |
| CA10-R | CGGACGAGCCTCTCCACTCT |
| CA6-F | GGAACTGGTTTTACTGATGCAGTG |
| CA6-R | TACAGGTTCAATACGGTGTTTGC |
| CA16-F | ATCCAGTAAGGATCCCAGACT |
| CA16-R | GATTTGCATAGTGGAGAGCAG |
| CVB3-F | AACGCCAAAACAACGGATGG |
| CVB3-R | GATCTGGGTCTGGGGGTAGT |
| EVD68-F | CGAGAGCATCATCAAAACAGCGACC |
| EVD68-R | CACTGTGCGAGTTTGTATGGCTTCT |
